# Functionalization of CD36 cardiovascular disease and expression associated variants by interdisciplinary high throughput analysis

**DOI:** 10.1371/journal.pgen.1008287

**Published:** 2019-07-25

**Authors:** Namrata Madan, Andrew R. Ghazi, Xianguo Kong, Edward S. Chen, Chad A. Shaw, Leonard C. Edelstein

**Affiliations:** 1 Cardeza Foundation for Hematologic Research/Department of Medicine, Sidney Kimmel Medical School, Thomas Jefferson University, Philadelphia, PA, United States of America; 2 Department of Quantitative and Computational Biosciences, Baylor College of Medicine, Houston, TX, United States of America; 3 Department of Molecular and Human Genetics, Baylor College of Medicine, Houston, TX, United States of America; 4 Department of Statistics, Rice University, Houston, TX, United States of America; University Medical Center Utrecht, NETHERLANDS

## Abstract

CD36 is a platelet membrane glycoprotein whose engagement with oxidized low-density lipoprotein (oxLDL) results in platelet activation. The CD36 gene has been associated with platelet count, platelet volume, as well as lipid levels and CVD risk by genome-wide association studies. Platelet CD36 expression levels have been shown to be associated with both the platelet oxLDL response and an elevated risk of thrombo-embolism. Several genomic variants have been identified as associated with platelet CD36 levels, however none have been conclusively demonstrated to be causative. We screened 81 expression quantitative trait loci (eQTL) single nucleotide polymorphisms (SNPs) associated with platelet *CD36* expression by a Massively Parallel Reporter Assay (MPRA) and analyzed the results with a novel Bayesian statistical method. Ten eQTLs located 13kb to 55kb upstream of the *CD36* transcriptional start site of transcript ENST00000309881 and 49kb to 92kb upstream of transcript ENST00000447544, demonstrated significant transcription shifts between their minor and major allele in the MPRA assay. Of these, rs2366739 and rs1194196, separated by only 20bp, were confirmed by luciferase assay to alter transcriptional regulation. In addition, electromobility shift assays demonstrated differential DNA:protein complex formation between the two alleles of this locus. Furthermore, deletion of the genomic locus by CRISPR/Cas9 in K562 and Meg-01 cells results in upregulation of CD36 transcription. These data indicate that we have identified a variant that regulates expression of *CD36*, which in turn affects platelet function. To assess the clinical relevance of our findings we used the PhenoScanner tool, which aggregates large scale GWAS findings; the results reinforce the clinical relevance of our variants and the utility of the MPRA assay. The study demonstrates a generalizable paradigm for functional testing of genetic variants to inform mechanistic studies, support patient management and develop precision therapies.

## Author summary

Platelets are anucleate cells that are best known as regulators of vascular hemostasis and thrombosis but also play important roles in cancer, angiogenesis, and inflammation. CD36 is a platelet surface marker that can activate platelet in response to oxidized low density lipoprotein (oxLDL). CD36 has been associated with numerous cardiovascular traits in human including blood lipid levels, platelet count, and cardiovascular disease prevalence in human genetic studies. Human variability in platelet CD36 levels are associated with the platelet response to oxLDL. However, the genetic mechanisms responsible for the variability of CD36 levels are unknown. We examined 81 genetic variants associated with *CD36* levels for functionality using a high-throughput assay. Of the ten variants that were identified in that assay, one doublet, rs2366739 and rs1194196, were confirmed using additional molecular and cellular assays. Deletion of the genomic region containing rs2366739 and rs1194196 resulted in overexpression of *CD36* in a cell culture system. This finding indicates a control locus which can serve as a potential target in modulating CD36 expression and altering platelet function in cardiovascular disease.

## Introduction

Cardiovascular disease (CVD) remains the number one cause of death globally [1]. Myocardial infarctions (MI) are acute events in CVD which are frequently the proximal causes of death or severe disability which are the result of platelet-rich thrombi [2]. Genome wide association studies (GWASs) have identified numerous common genetic variants associated with the risk of CVD and platelet function parameters, but these variants are usually not causative due to the resolution of the genotyping platforms used and genetic linkage. One of the genes identified by GWAS as associated with platelet count, lipid levels. and CVD is the platelet oxidized LDL (oxLDL) receptor, CD36 [3–5].

CD36 is a transmembrane protein belonging to the class B scavenger receptor family expressed in platelets and variety of other cells [6–8]. It binds to many ligands such as oxidized phospholipids (oxPL) and oxidized low-density lipoprotein (oxLDL) long-chain fatty acids [9]. In platelets, CD36 interaction with oxLDL and thrombospondin-1 (TSP1) triggers MAP and Src family kinase dependent signaling events leading to platelet activation, [10, 11] which also lead to increase in P-selectin expression and αIIbβ3 activation [10]. Deletion of CD36 in mice fed a high fat diet results in attenuation of the pro-thrombotic state and platelet hyper-activity [10].

CD36 deficiencies have been identified which result in increased risk of cardiomyopathy, hyperlipidemia and insulin resistance [12–15]. In type I deficiency, monocytes and platelets lack CD36 expression, whereas in type II only platelets lack CD36 expression. CD36 deficiency is more frequent in black and Asian populations. Our platelet transcriptomic data also show that platelet *CD36* RNA levels are lower in the black population and in women [16, 17]. The molecular mechanisms behind *CD36* deficiency have been attributed to variants causing defects in protein maturation or frameshift, resulting in an absence of protein [14, 18].

Among subjects without CD36 deficiency, there is a wide range of platelet CD36 surface expression and the level of CD36 correlated with reactivity to oxLDL [19]. Many genetic variants have already been reported to be associated with platelet CD36 expression, however, these variants span a large linked genomic area and no functional analysis has been carried out [19, 20]. We have previously reported platelet expression Quantitative Trait Loci (eQTLs) that associate single nucleotide polymorphisms (SNPs) with platelet RNA levels, indicating genetic variability effecting gene expression [21]. *CD36* is one of the 612 platelet-expressed RNAs whose abundance has significant genotypic associations. 81 eQTL SNPs located within a +/- 100kb window of the CD36 gene are associated with platelet CD36 mRNA levels at a significance of P<1x10^-6^, spanning a range of 118kb.

We hypothesized that a parallel screening method would be more efficient and cost-effective to identify causal variants instead of a one-at-a-time approach. We used a massively parallel reporter assay (MPRA) to screen the 81 platelet eQTLs associated with *CD36* mRNA. We developed new statistical methods for MPRA analysis, and we were able to identify rs2366739 and rs1194196 as functional variants that alter transcriptional regulation. We further tested these MPRA-functional variants, showing significant transcription shift between the reference and alternate alleles by luciferase assays, electromobility shift assays (EMSA) and using CRISPR/Cas edited stable cell lines. Finally, we used the Phenoscaner GWAS aggregation tool to reinforce the clinical relevance of our functional variants. Using these approaches, we have identified genetic variants that modulate platelet CD36 expression and have clinical associations.

## Results

### Landscape of CD36 variants

**Fig 1:**
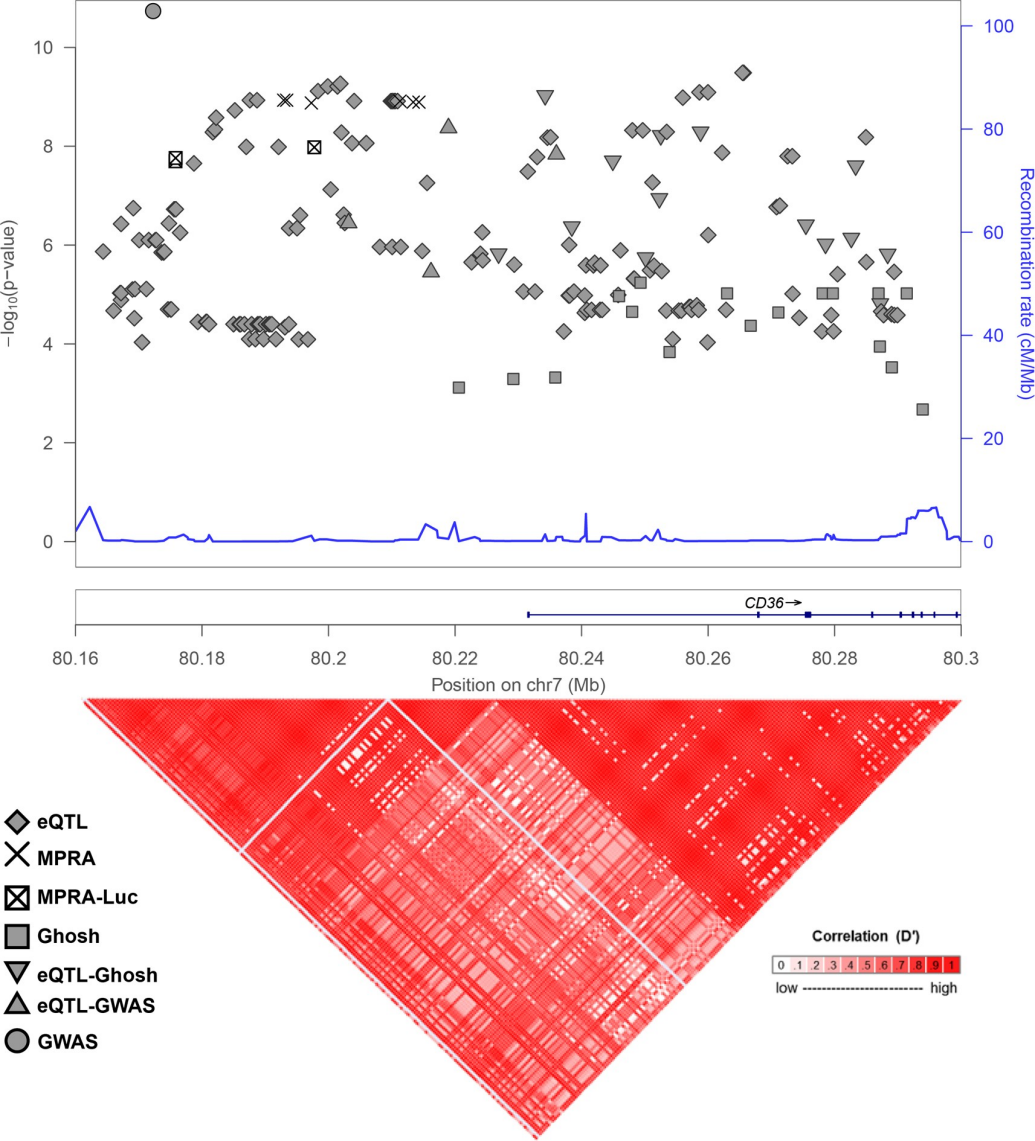
Genomic context of *CD36* variants. (Upper) Manhattan plot of *CD36* variants identified as platelet eQTLs only (diamonds), associated with CD36 surface levels (squares), GWAS-implicated (circle), eQTLs and CD36 surface level associated (upside down triangle), eQTLs and associated with GWAS-implicated (triangle), eQTLs and functional in MPRA assay (X’s), or eQTLs functional in MPRA assay and validated by luciferase (squared X).–log_10_ P-values are significance of eQTL association, if available, or from CD36 surface level association. GWAS-implicated variant (circle) is associated with platelet count (P = 2 x 10^−17^) and vertical position does not represent significance. (Lower) LD-plot of genomic locus with D’ values calculated with 1000 genomes JPT (Japanese) population.

We have previously published the results of a cis-eQTL analysis of platelet gene expression [21]. SNPs found to be associated with platelet *CD36* mRNA expression are indicated by diamonds on the Manhattan plot in Fig 1. Ghosh et al. have also looked for associations between *CD36* SNPs and CD36 protein expression [19]. SNPs identified in that report are indicated in Fig 1 by squares, and SNPs that were identified both by our eQTL study and Ghosh et al. are indicated by upside-down triangles. Several *CD36* SNPs have been identified in GWAS studies to associate with platelet count or volume. The GWAS-identified SNPS rs6961069, rs13236689, rs2177616, and rs11764390 are also platelet *CD36* eQTLs and are indicated by triangles in Fig 1 [4, 22, 23]. rs139761834 (Fig 1, circle) was identified by GWAS but not by eQTL analysis [23]. The genomic region containing these variants encompasses the 5’ end of the *CD36* gene and upstream sequence and is highly linked as indicated by the linkage disequilibrium plot in the bottom panel of Fig 1. Given that most data on CD36 deficiency and expression has been obtained from Japanese subjects, D’ values were calculated using the 1000 Genomes JPT population. This strong linkage makes identification of the functional variant difficult, and therefore functional analysis requires experimental testing of individual SNPs. The large genomic span and the number of *CD36* phenotype-linked variants necessitated a highly parallel approach for functional testing.

### Massively parallel reporter assay identifies CD36 SNPs with allelic difference in CD36 gene expression

**Fig 2:**
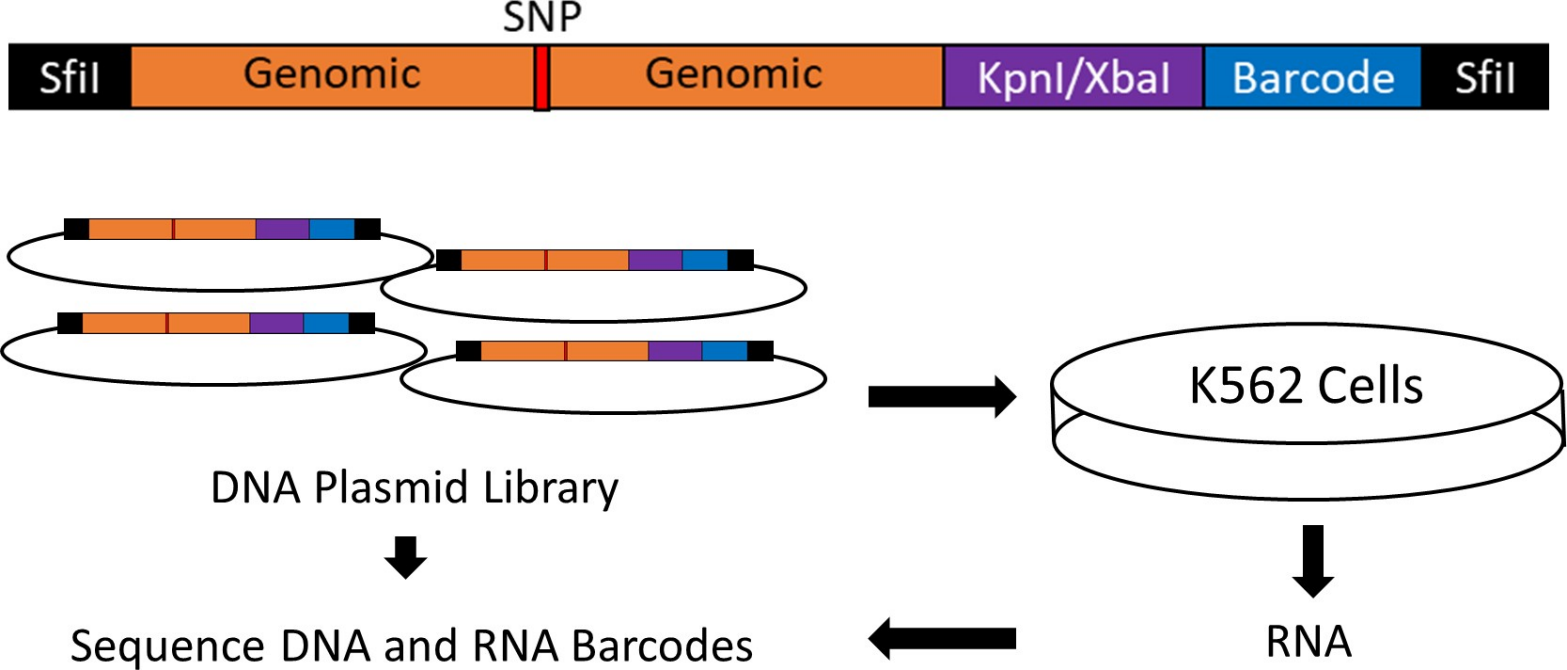
Design of the massively parallel reporter assay. A library of oligonucleotides was designed and synthesized containing *CD36* eQTL SNPs surrounded by 150bp of genomic context 5’ to a KpnI/XbaI linker used to insert a minimal promoter-luciferase cassette derived from pNL3.2. 3’ to the linker is a unique 10bp barcode. SfiI restriction sites generated by PCR were used to clone the oligo into the pMPRA1 backbone vector. The plasmid library was transfected into K562 cells and 48 hours RNA was harvested. Barcodes from the harvested RNA and plasmid DNA library was quantified by NGS.

We generated a library of plasmids in which a unique 10bp barcodes located downstream to a luciferase cassette were transcribed under the control of a 150bp genomic fragment containing a *CD36* platelet eQTL SNP. Each allele of each variant is associated with 40 unique barcodes in order to give high statistical power for detecting variant function despite variation in the NGS outputs. Successful conduction of a MPRA experiment requires high transfection efficiency to allow for sufficient expression of barcode diversity. We compared the RNA-seq gene expression profiles of several hematopoietic cell lines (derived from ArrayExpress (https://www.ebi.ac.uk/arrayexpress/) accession number E-MTAB-4101) to RNA-Seq data from cultured megakaryocytes (derived from Blueprint Epigenome Data [24]). All comparisons were significant at P<0.0001 and the Spearman correlation coefficient ranged from 0.658 to 0.720 (Table 1). We ultimately chose to utilize K562 cells due to their myelogenous origin, transfection efficiency, the comparable expression profile similarity to other cell lines and megakaryocytes, and their prior successful use in MPRA experiments [25]. Transcription activity driven by each variant was then measured by quantifying RNA barcodes output after they have been “normalized” or jointly modeled against the plasmid DNA library barcode inputs. An overview of MPRA protocol is presented in Fig 2.

**Table 1:** Comparison of cell line gene expression to megakaryocytes.

Cell Line	Spearman Correlation Coefficient
F36P	0.688
HEL9217	0.720
K562	0.658
KU812	0.710
MEG01	0.709
UT7	0.682

**Fig 3:**
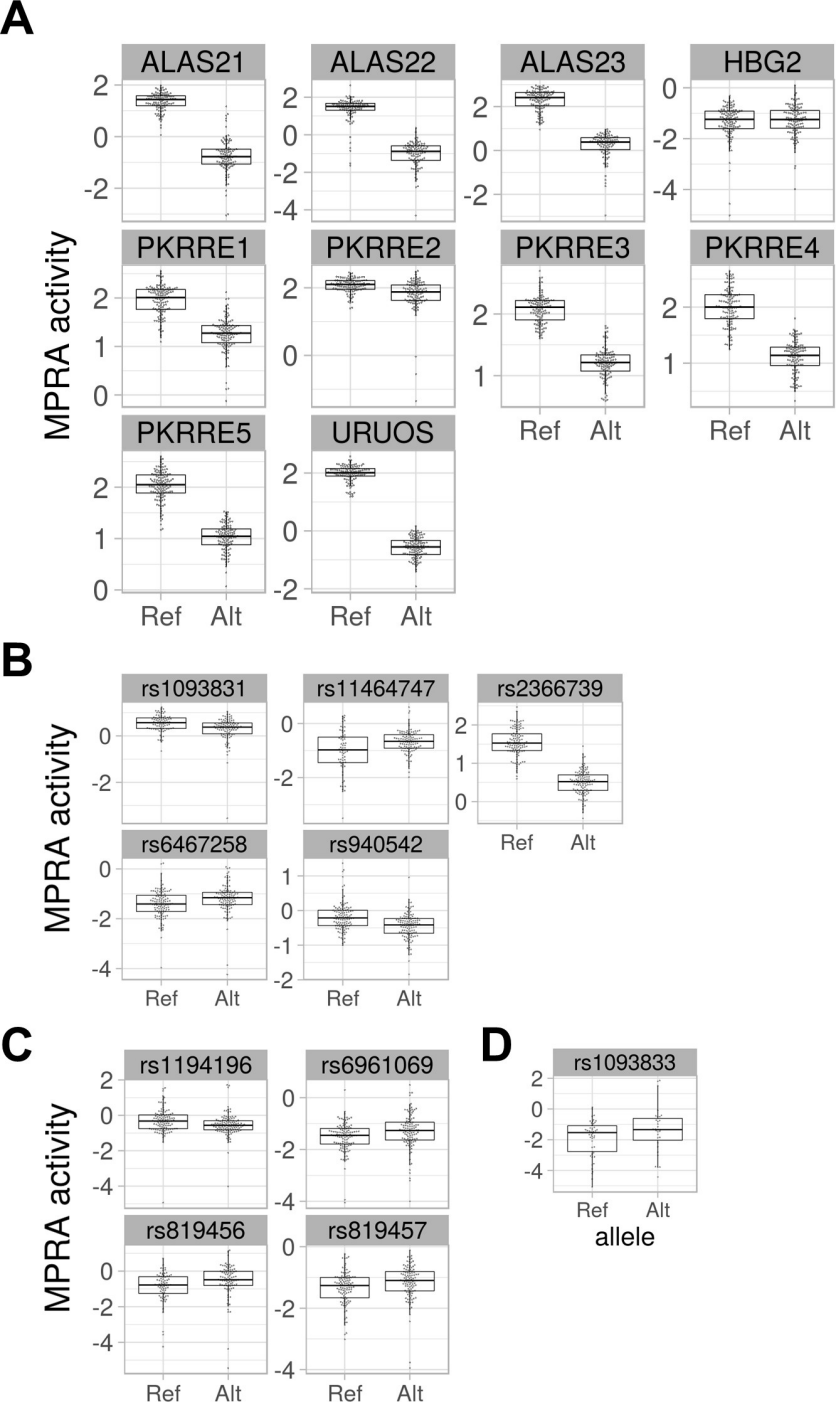
Results of MPRA assay of *CD36* eQTL SNPs. (A) MPRA activity of positive control constructs. (B) MPRA activity of *CD36* eQTL SNPs with significant transcription shifts identified by T-Test. (C) Additional *CD36* variants with significant transcription shifts identified by U-Test. (D) Additional *CD36* SNP with significant MPRA transcription shift identified by Bayesian analysis.

We employed two statistical methods to analyze the result of the MPRA: a traditional method that involves computing variant activities defined by a ratio transformation of mRNA to DNA input and a second Bayesian method. The traditional method normalizes the counts for sample depth, removes barcodes with low representation in the plasmid library, and computes the activity as $log\left(\frac{mRNA}{DNA}\right)$ of each barcode in each allele in each transfection, and then uses a t-test and false discovery rate correction to compare the mean activity levels of alleles for each SNP. This approach yields 14 hits with Q < .05, including nine of the 10 controls (Table 2; Fig 3A and S1 Fig) and five CD36 SNPs: rs2366739, rs940542, rs1093831, rs11464747, and rs6467258 (Table 2; Fig 3B and S1 Fig). MPRA activities can occasionally defy the normality assumption underlying the t-test,[26] so we also employed the non-parametric Mann-Whitney U-test, under the same frequentist methodologic paradigm as the t-test approach. Analyzing the transcription activity level with a U-test revealed an additional four significant variants, rs1194196, rs6961069, rs819456, and rs819457 (Table 2; Fig 3C and S1 Fig).

**Table 2:** MPRA-functional *CD36* variants.

Control/CD36 SNP	Transcription Shift	t-test		u-test		Bayesian Model	
		P-Value	Q-value*	P-Value	Q-Value*	Posterior Mean	95% Credible Interval^†^
**URUOS**	-2.55525	1.94E-131	**1.76E-129**	2.08E-38	1.89E-36	-2.44298	**-2.616 : -2.267**
**PKRRE5**	-1.00354	9.86E-73	**4.49E-71**	1.41E-37	6.40E-36	-1.11463	**-1.282 : -0.944**
**PKRRE3**	-0.87213	1.24E-71	**3.77E-70**	4.44E-37	1.35E-35	-1.01771	**-1.183 : -0.848**
**ALAS23**	-2.08592	8.95E-62	**2.04E-60**	5.82E-33	8.82E-32	-2.00429	**-2.276 : -1.703**
**ALAS22**	-2.413	2.53E-57	**3.83E-56**	1.11E-28	1.12E-27	-2.48037	**-2.838 : -2.114**
**ALAS21**	-2.10623	6.23E-57	**8.10E-56**	1.52E-32	1.97E-31	-2.00922	**-2.33 : -1.699**
**PKRRE4**	-0.89499	2.37E-56	**2.70E-55**	7.73E-36	1.76E-34	-0.93448	**-1.097 : -0.769**
**PKRRE1**	-0.69606	7.38E-39	**7.46E-38**	2.57E-30	2.92E-29	-0.75478	**-0.924 : -0.59**
**PKRRE2**	-0.26871	1.76E-06	**1.45E-05**	1.47E-08	1.34E-07	-0.37922	**-0.698 : -0.055**
**HBG2**	0.06142	4.96E-01	**7.28E-01**	7.87E-01	8.97E-01	-0.01277	**-0.231 : 0.214**
**rs2366739**	-1.04532	6.87E-59	**1.25E-57**	1.41E-35	2.57E-34	-1.13049	**-1.295 : -0.955**
**rs940542**	-0.2785	2.56E-07	**2.33E-06**	6.51E-07	5.39E-06	-0.29285	**-0.481 : -0.12**
**rs1093831**	-0.273	1.07E-04	**8.09E-04**	7.70E-05	5.84E-04	0.10111	**-0.113 : 0.315**
**rs6467258**	0.22024	6.72E-03	**4.37E-02**	7.47E-04	5.23E-03	-0.05448	**-0.259 : 0.16**
**rs1194196**	-0.23346	1.21E-02	6.45E-02	1.07E-03	**6.93E-03**	-0.08582	**-0.271 : 0.111**
**rs11464747**	0.36056	1.02E-03	**7.16E-03**	1.70E-03	1.03E-02	1.23996	**0.962 : 1.518**
**rs819456**	0.31541	3.05E-02	1.32E-01	2.68E-03	**1.52E-02**	0.46373	**0.079 : 0.861**
**rs6961069**	0.21993	1.14E-02	6.45E-02	2.90E-03	**1.55E-02**	0.01234	**-0.211 : 0.233**
**rs819457**	0.18773	1.58E-02	7.58E-02	3.07E-03	**1.55E-02**	0.14833	**-0.072 : 0.372**
**rs1093833**	0.54135	1.04E-01	3.03E-01	1.23E-01	3.12E-01	1.00913	**0.271 : 1.764**

* Significant Q-values are highlighted (Q<0.05).

†Significant credible intervals are highlighted (Interval does not contain 0).

We sought to avoid two statistical limitations that lead to information and power loss in the MPRA experiment using the traditional analysis approach. First, transforming the data with a ratio removes the ability to model systematic effects of the DNA and RNA libraries. Second, discarding barcodes with low or 0 counts discards data that may be informative. Therefore, we also employed a Bayesian count model of the data generating process that models the NGS reads of each barcode observed from sequencing the plasmid library and from each transfection experiment as arising from coupled negative binomial distributions. The means of the negative binomial distributions are proportional to the depth of the sequencing of each sample and, in the case of RNA samples, the mean of the barcode’s DNA read count. Empirical gamma priors on the negative binomial parameters were estimated marginally across all SNPs in the assay. The log difference in the depth- and DNA-normalized RNA means gives a quantity comparable to the difference in mean activity (i.e. the ratios) analyzed under the t-test-based method. Thus, this model provides a posterior on transcription shift for each SNP after directly accounting for more sources of variation and more data from the MPRA experiment than the traditional approach. We identify a SNP in question as a functional hit if a 95% credible interval for the posterior distribution of the transcription shift excludes 0. This process yields 19 hits, including the same nine of the ten controls and ten CD36 SNPs, the nine listed above plus an additional variant, rs1093833 (Table 2; Fig 3D and S1 Fig) that was not identified by the frequentist approaches. As shown in Fig 1, (MPRA positive hits indicated by X’s) these SNPs are in high LD with one another, indicating close physical proximity in what is likely the regulatory region of the *CD36* gene. The complete analysis results of the tested MPRA variants and controls is given in S1 and S2 Tables.

### Validation of differential enhancer activity of rs2366739-rs1193196 locus

**Fig 4:**
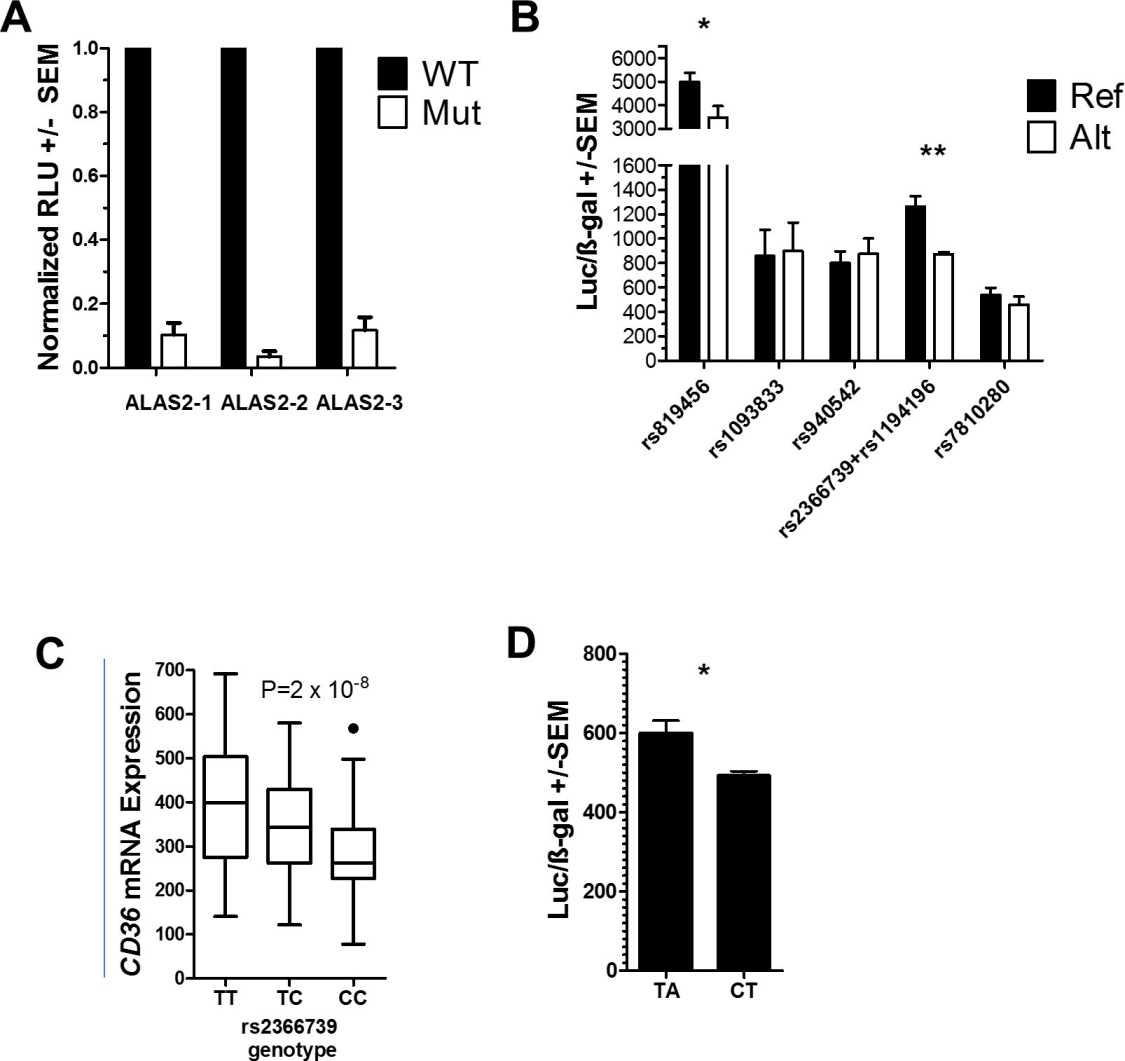
Luciferase assay of MPRA-identified variants. (A) Relative luciferase activity of reporter vectors containing control (WT) or mutated (Mut) GATA1 sites. (N = 3) Significance by one-sample T-test (B) Luciferase activity normalized to β-gal activity of MPRA-functional *CD36* variants in K562 cells. Reference (Ref) and Alternate (Alt) alleles for each variant is indicated. (C) Platelet eQTL analysis indicated that platelet *CD36* mRNA levels are associated with rs2366739 genotype (P = 2 x 10^−8^). Line = mean expression, Box = 25^th^ to 75 interquartile range (IQR), Whiskers = 1.5 x IQR. (D) Luciferase activity normalized to β-gal activity of reporter vectors containing the rs2366739-rs1194196 locus transfected into Meg-01 cells. (B,D) N = 3 to 5 Significance by two-sample T-test. * <0.05, ** < 0.01.

To verify the transcription shifts identified by the MPRA, we tested three of the controls, the two most significant MPRA hits by t-test (rs2366739 and rs940542), the additional two most significant MPRA hits identified by U-test (rs1193196 and rs819456), and the additional significant MPRA hit identified by Bayesian analysis (rs1093833) by reporter assay. Reporter plasmids containing reference or alternate alleles of the eQTLs were transfected into K562 cells and assayed after 48 hours for luciferase and β-gal expression. We first investigated the activity of each control sequence (ALAS2-1, 2 and 3) containing original or disrupted GATA1 binding site. As predicted and shown previously the sequences with original binding site exhibited more enhancer like activity by luciferase expression than the sequence with disrupted (Alt) binding site (Fig 4A) [25].

Because eQTLs rs2366739 and rs1194196 are within 21 base pairs of each other and in high linkage disequilibrium we constructed single oligos which contains either reference (T-A for rs2366739-rs1194196) or alternate alleles (C-T) for both eQTLs. These genotypes account for 96% of the observed haplotypes from all populations. Out of the five tested constructs, rs819456 and rs2366739-rs1194196 showed significant transcriptional difference between their reference vs alternate allele as depicted by the luciferase levels (Fig 4B). The rs2366739-rs1194196 results are in agreement with our platelet eQTL data and whole blood eQTL data from Jansen et al. that indicate that the ‘C’ allele of rs2366739 is associated with lower levels of *CD36* mRNA (Fig 4C) [21, 27]. Overall expression from the rs819456 constructs was higher than other constructs but the direction of the difference between alleles (higher in the reference, Fig 4B) was opposite to that of the MPRA results (lower in the reference (Fig 3B)). Finally, to test the transcriptional activity in a system more closely related to megakaryocytes, we repeated the luciferase assay of the rs2366739-rs1194196 construct in Meg-01 chronic myelogenous leukemia cells. In this system, the difference in transcriptional potency between the two alleles was consistent with the results in K562 cells showing an approximate 20% reduction in transcription between the two alleles (Fig 4D).

rs2366739 has been described as an CD36 eQTL not just in our platelet data but in whole blood data from Võsa et al. (P = 1.3 x 10^−267^) [28]. In addition rs2366739 has been associated with DNA methylation levels (P = 6.51 x 10^−81^) [29]. These results in addition to the high significance and directional agreement of the rs2366739-rs1194196 luciferase and MPRA results lead us to pursue the rs2366739-rs1194196 locus in further tests.

### Differential protein binding between reference and alternate allele of rs2366739 and rs1194196

**Fig 5:**
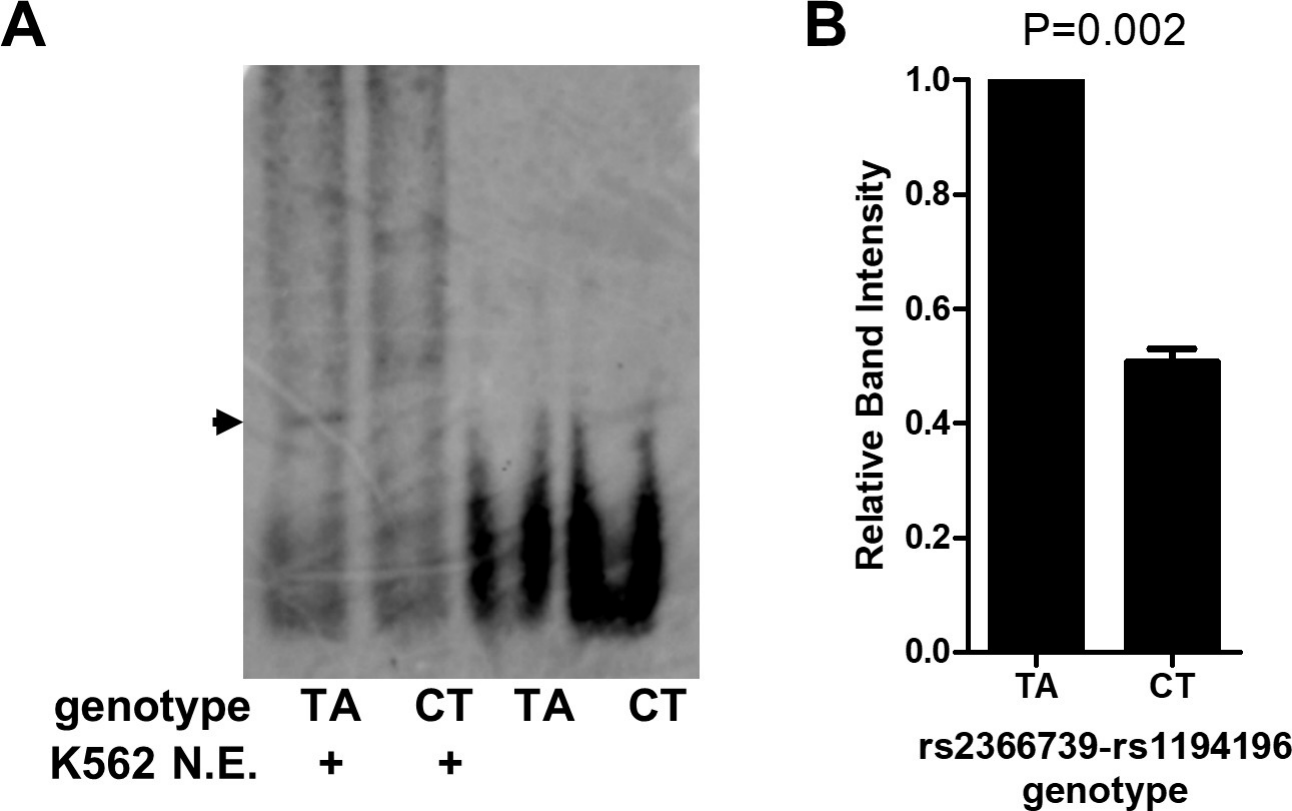
rs2355739-rs1194196 variants alter protein:DNA interactions. (A) Electromobility shift assay using probes containing 70bp of genomic sequence containing SNPs rs2355739 and rs1194196. Digoxin-labeled Probes were incubated with K562 cell nuclear extract where indicated, resolved by gel electrophoresis, and imaged with anti-digoxin antibodies. Arrow indicated specific complex formed by protein:DNA interactions. (B) Quantification of DNA:protein complex by densitometry. (N = 3) Significance by one-sample T-test.

One of the mechanisms by which gene expression is regulated is the binding of transcription factors to regulatory elements. To test if the difference in transcription between TA and CT alleles of the rs2366739-rs1194196 constructs is due to alteration in transcription factor binding affinity, we performed an electrophoretic mobility shift assay (EMSA) to compare binding of K562 nuclear extracts to probes derived from the two different haplotypes. The results show formation of a DNA:protein complex with twice as much affinity to the TA genotype probe than to the CT genotype probe (Fig 5).

### In vivo validation of rs2366739-rs1194196 locus as CD36 regulatory element

**Fig 6:**
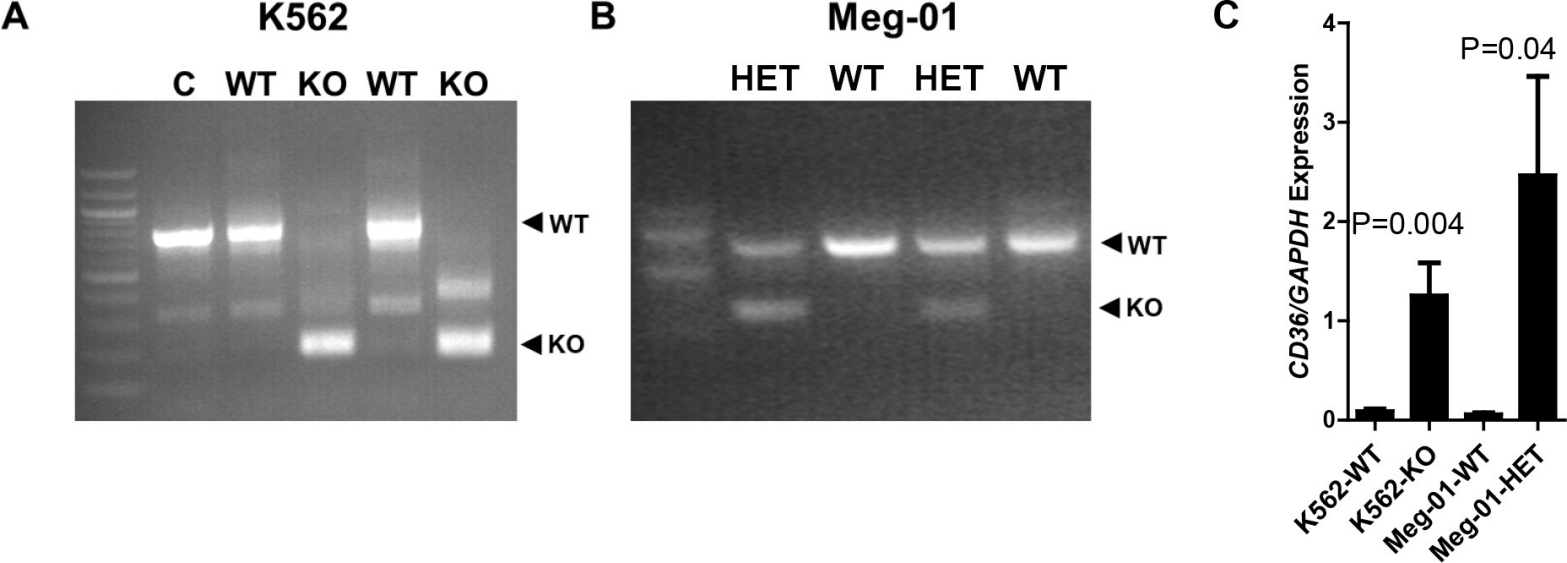
Deletion of the rs2355739-rs1194196 locus increased *CD36* expression. Deletion of the rs2355739-rs1194196 locus in (A) K562 and (B) Meg-01 cells by CRISPR/Cas9 was validated by PCR using primers flanking the deleted section. WT clones did not contain a deletion while KO clones contained the deletion. (C) qRT-PCR of *CD36* mRNA indicated that the wildtype (WT) K562 and Meg-01 clones contained less *CD36* RNA than KO clones lacking the rs2355739-rs1194196 locus (KO).

To confirm the locus containing rs2366739 and rs1194196 regulates CD36 expression, we generated K562 cell lines with deletion of 573 basepairs containing this region using CRISPR/Cas9. The deletion was confirmed with PCR comparing clones transfected with sgRNAs to those transfected with vectors with no sgRNA (Fig 6A, lane C). Clones labeled WT do not contain a deletion whereas clones labeled KO were successfully altered. To determine the effect of this deletion on *CD36* RNA expression, CD36 transcript levels were measured by qRT-PCR. In the cells with the rs2366739-rs1194196 locus removed, *CD36* mRNA was ~13 times greater than the clones with the region intact (Fig 6C). We also analyzed the effect of this deletion in Meg-01 cells. We were only able to obtain clones containing heterozygous knockouts (Fig 6B). However, like in K562 cells, this resulted in a ~39-fold increase in *CD36* mRNA levels (Fig 6C). This supports the evidence that this genomic region identified by MPRA regulated expression of the *CD36* gene.

### Assessment of clinical significance

To assess the clinical relevance of our findings we used the PhenoScanner tool, which aggregates large scale GWAS findings. We re-identified the rs2366739 variant in the CD36 associations and we found it to be strongly associated with platelet volume and count but not with phenotypes unrelated to platelet activity. These results reinforce the clinical relevance of our variants and the utility of the MPRA assay.

## Discussion

The expression of CD36 has been actively studied since the identification of a deficiency in healthy Japanese and US donors [30]. CD36 deficiency has been divided into two types: Type I in which neither platelets nor monocytes express surface CD36 and Type II in which only platelets lack expression [31]. Lack of CD36 leads to numerous cellular phenotypes including defective uptake of long chain fatty acids by the myocardium[32, 33] and altered lipid profiles [34, 35]. Lack of CD36 has also been associated with altered foam cell formation in both humans and mice [36, 37]. Even in non-deficient patients a wide range of CD36 expression has been observed and this variation has been associated with the platelet response to oxLDL [19, 38].

Five genetic causes of type I deficiency have been identified, two of which lead to alterations in post-translation modification or surface trafficking, the other three which lead to frame-shift mutations [18, 39–41]. The basis of type II deficiency remains unclear. Mechanisms behind the broad range of non-deficient expression has been explored previously. For example Ghosh et al. previously identified a number of SNPs that are associated with platelet CD36 levels and Masuda explored surface levels in individuals heterozygous for the deficiency mutations mentioned above [19, 38]. However, given the tightly linked nature of the locus (Fig 1), neither of these studies identified functional variants.

Our data presented here represents the first comprehensive analysis and testing of genetic variants associated with *CD36* mRNA expression. We tested 81 variants associated with platelet *CD36* mRNA levels, some of which overlap with variants previously associated with surface levels, by MPRA. Of these variants, rs2366739 and rs1194196, which are within 21 base pairs of each other, were identified by MPRA and validated by (1) altering expression of a linked reporter gene, (2) altering protein binding in a gel-shift assay, and (3) altering endogenous *CD36* expression when the locus containing these variants was deleted by genome editing. Importantly, rs2366739 has previously been associated with both platelet count (P = 9.13 x 10^−10^) and platelet volume (P = 5.27 x 10^−11^) [22]. Taken together, we found that the MPRA analysis refined the region of CD36 responsible for gene expression and within this region the MPRA distinguished specific GWAS hits for platelet-associated phenotypes. This result emphasizes the utility of the MPRA to clarify and refine association analyses in highly linked regions.

The analysis methods employed for our CD36 MPRA are another important contribution of this work. We applied t- and U-tests that have been employed in prior MPRA studies, but we also introduced a new and more comprehensive Bayesian approach. Our new method recapitulates the findings of the prior methods, but also identifies a variant that was missed by the prior approaches. A key advantage of our new statistical method is a generative stochastic model that probabilistically accounts for the discrete nature of NGS count data as well as sources of variation in the MPRA experiment without having to discard zero counts. S4 Fig shows a Kruschke diagram of the generative process considered by the model. The sources of variation addressed include variation in the barcode abundances in the cDNA library as well as other factors. Furthermore, the use of empirical priors provides estimate shrinkage; noisy parameter estimates are shrunken towards more moderate levels observed throughout the rest of the assay. This process helps eliminate false positives without the heavy statistical burden of multiple testing correction procedures like false discovery rates. We have previously studied the statistical power of MPRA experiments using the standard approaches [26]. Although the prior methods were at least partially successful to analyze MPRA data, our Bayesian model appears both practical and more powerful. Therefore, our paper demonstrates effective statistical improvements to analysis of MPRA studies. This approach may be particularly important in larger scale genome wide MPRA studies, and more work is warranted to improve the cost-effectiveness as well as the discovery potential of MPRA assays.

One of the weaknesses of this study is that we have been unable to identify the DNA-binding protein factor whose binding is altered by the variants. The rs2366739-rs1194196 is located in a genomic locus that contains ChIP-Seq signals for Histone H3K27 acetylation and is in a DNA hypersensitive region in primary and cultured hematopoietic stem cells.[42] However ChIP-Seq data of specific transcription factors in CD34 cells and megakaryocytes is limited and we have not been able to identify a sequence-specific factor bound to the region. We have analyzed the sequence using the transcription factor binding prediction algorithm JASPAR [43] and found predictions for the well-studied megakaryocytic transcription factors GATA1/2/3 and Gfi1/Gfi1b on the TA haplotype but not CT.[44, 45] Interestingly these two transcription factor families, GATA and Gfi1, have opposite functions; GATA factors are transcriptional activators while Gfi1 and Gfi1b are transcriptional repressors. This suggests that these two factors may regulate *CD36* expression in a complex manner that is affected by genotype.

The dual nucleotide changes assayed in Figs 4 and 5 indicate that the TA haplotype results in higher transcriptional activity and an enhancement of protein binding, suggesting that a positive regulatory factor is more readily bound to the TA haplotype. However, deletion of the locus resulted in enhanced *CD36* expression in both K562 and Meg-01 cells, suggesting a negative regulatory activity also exists. There are differences between these experiments which can contribute to understanding our observations: The luciferase and EMSA experiments are performed *in vitro* using 70bp of genomic sequence surrounding rs2366739- rs1194196, either by itself as an EMSA probe or cloned into a luciferase vector. The genomic deletion is a 573bp deletion of DNA in a genomic chromatin context. In the genomic context, the locus is located 56 kbp and 92 kbp from the two transcriptional start sites of *CD36*. In the plasmid context, the transcription start site is immediately downstream from the cloned fragment. We hypothesize that additional negative regulatory factors bind to genomic DNA, outside the limited context used in MPRA and the 70bp fragment tested by luciferase and EMSA (S2 Fig). Therefore, in experimental conditions containing only the 70bp region, a positive regulatory factor determines transcriptional/binding activity. But in the genomic context, deletion of the 573bp fragment results in the loss of both positive and negative factors, resulting in a net increase in transcription, perhaps driven by enhancers more proximal to the promoter. More work is required to further investigate this hypothesis.

Currently, more human genetic studies are moving beyond associations of genotypes with phenotypes to seeking the molecular mechanisms behind the variants responsible for the observed traits. Mechanistic understanding of genetic variants will provide better understanding of the observed physiology, allow for more precise biomarkers, and identify potential new therapeutic targets. Given the large number of variants potentially associated with a trait in highly linked genomic regions such as *CD36*, high-throughput methods are necessary to efficiently test and identify functional polymorphisms in an unbiased manner. Our identification of the genetic locus responsible for inter-individual variation in non-deficient *CD36* expression opens new areas of investigation into the link between this locus and platelet function, serum lipid levels, and atherosclerosis.

## Materials and methods

### Ethics statement

K562 and Meg-01 cells were obtained from the American Tissue Culture Collection (ATCC)

### Cell culture

K562(CCL-243) and Meg-01 (CRL-2021) cells from ATCC were maintained in RPMI 1640 media (Invitrogen, CA 10-040-cv) containing penicillin-streptomycin and 10% FBS.

### MPRA

**Fig 7:**
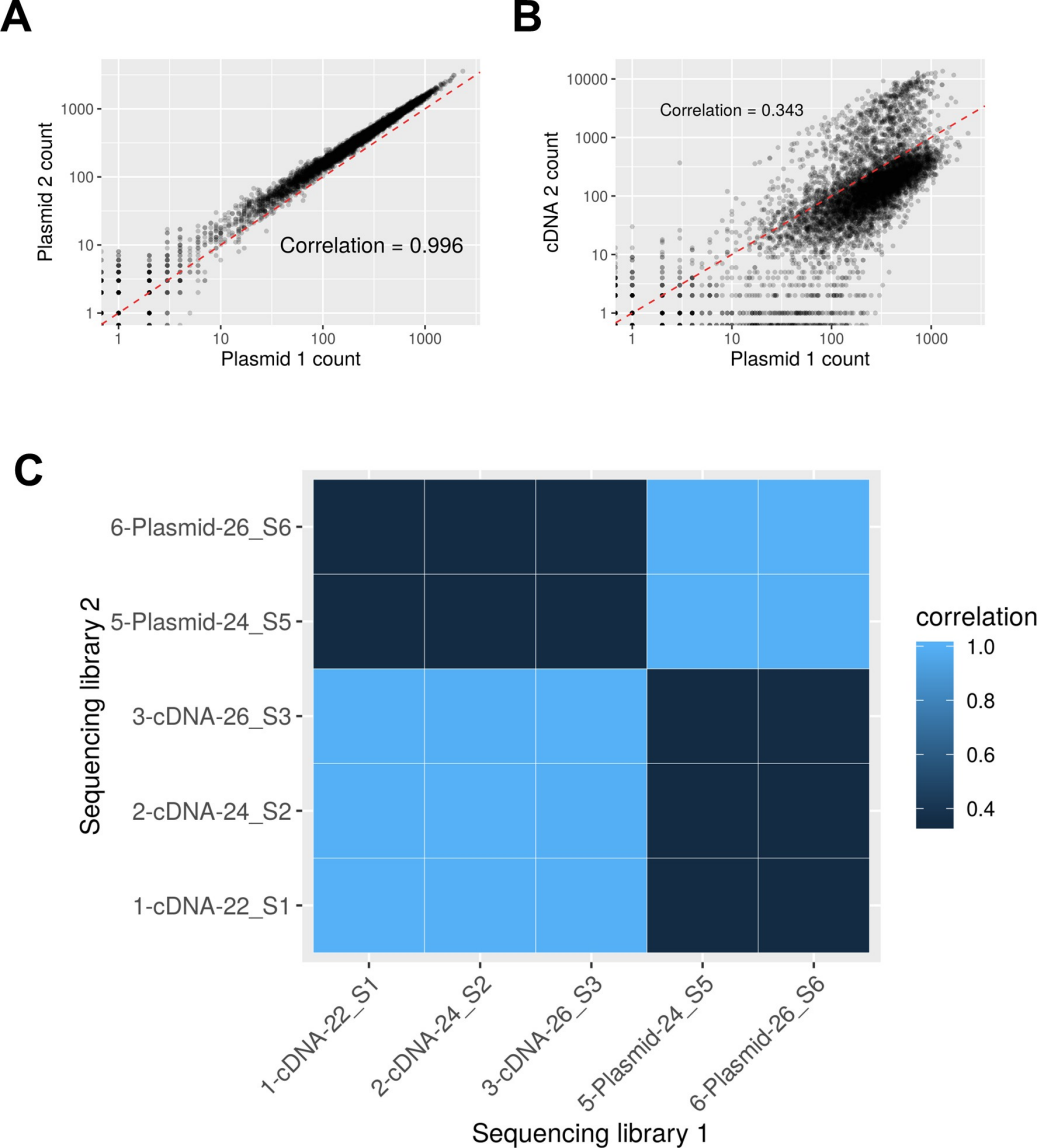
Correlation of sequencing libraries. (A) Scatterplot of barcode counts showing the correlation between the two sequencing samples prepared from the plasmid library. (B) A similar plot showing the expectedly lower correlation between one plasmid sample and a cDNA sample. Because the barcodes need to go through a transcription step to be present in the cDNA sample, these counts are inherently more noisy and less correlated with their plasmid barcode inputs. C) A heatmap showing the pairwise correlation values between all five samples in the experiment.

The MPRA design was based on the method previously published (A graphical summary is presented in Fig 2) [46]. To design the *CD36* MPRA library, we used a p-value threshold of p < 1x10^-6^ to select the expression quantitative trait loci (eQTLs) most highly associated with *CD36* expression in the PRAX study surrounded by 150bp of hg38 genomic context [21]. After discarding 5 eQTLs that contained digestion sites for restriction enzymes used in the library preparation in their genomic context, this yielded a set of 81 CD36 SNPs to assay. We also included 10 SNPs previously identified in the literature as directly affecting *ALAS2*, *HBG2*, *PKRRE*, and *URUOS* expression as a set of positive controls (S3 Table) [47, 48]. We synthesized 40 oligonucleotide replicates per allele(Agilent, Santa Clara, CA), each uniquely tagged with inert 10bp barcodes which followed the design criteria stipulated in Melnikov et al. [49]. The library of oligonucleotides was amplified using emulsion PCR and the primers 5’-TGCTAAGGCCTAACTGGCCAG-3’ and 5’-CTCGGCGGCCAAGTATTCAT-3’ which also added additional sequence containing SfiI restriction enzyme sites to each end. After each oligo was directionally cloned into pMPRA1 (Addgene, Cambridge, MA) using the SfiI sites, a minimal promoter and luciferase cassette derived from pNL3.2 (Promega, Madison, WI) was inserted in between the genomic sequences and the barcode using KpnI and XbaI sites. This library of plasmids was transfected into K562 cells and then RNA was harvested 48 hours later. After reverse transcription with a polyT primer, three separate amplifications of the cDNA were performed to generate RNA sequencing libraries. The barcodes contained in the MPRA plasmid library were subjected to two separate amplifications to generate DNA sequencing libraries. RNA and plasmid barcode expression was quantified by next generation sequencing on an Illumina MiSeq in the Children’s Hospital of Philadelphia sequencing core. After extraction from the fastq files, barcodes with a quality of Q>30 at every base was counted. RNA barcode counts were analyzed in conjunction with the DNA barcode counts to control for variances in barcode abundances introduced by library generation. Quality control data concerning the sequencing libraries are presented in S4 Table and Fig 7 and S3 Fig.

Both frequentist (t-tests and U-tests) and Bayesian analyses were applied to identify variants that caused alterations to transcription activity by use of RNA-to-DNA ratios to examine allelic differences. Analysis of the barcode counts proceeds by computing the MPRA activity of each barcode as the log-ratio of the depth-normalized RNA counts to depth-normalized DNA counts and each variant’s corresponding “transcription shift”. The transcription shift of a variant is defined as the difference in activity between the alternate and reference alleles.

**Fig 8:**
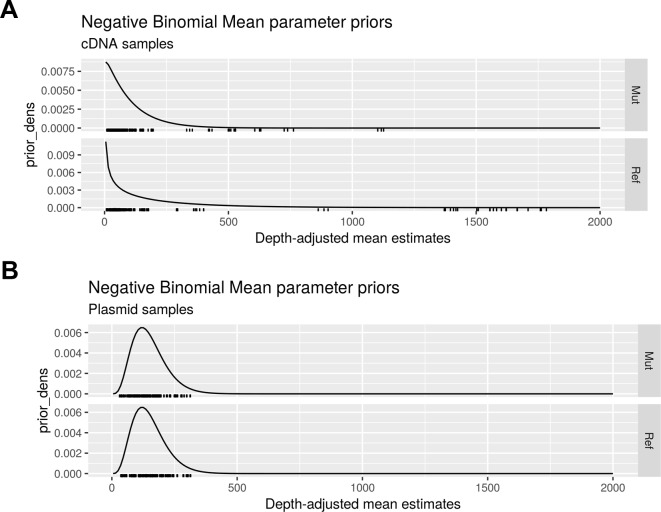
Negative binomial mean parameter priors. Density functions of the empirically estimated gamma priors for (A) the cDNA samples and (B) the plasmid library samples.

The application of traditional frequentist tests to MPRA activities has been previously described [25, 26]. The novel Bayesian analysis models the barcode count data using negative binomial distributions with empirical gamma priors (Fig 8), this analysis method provides greater sensitivity than traditional methods while retaining specificity.

### Luciferase assay

We designed and ordered oligonucleotides (IDT-DNA, Coralville, IA) containing the candidate MPRA-functional SNPs and 40bp of flanking genomic sequence (S5 Table). The amplified sequence was inserted into nano-luciferase containing plasmid pNL3.2 (Promega, Madison, WI) using HindIII and XhoI (ThermoFisher, Waltham, MA) restriction sites. The sequence was confirmed by sequencing. Three independent preparations of reporter plasmids and β-gal expression plasmids were cotransfected in K562 cells or Meg-01 cells and luciferase assay was carried out after 48 hours using Nanoglo luciferase kit (Promega) and normalized to ß-gal expression measured using assay reagent (ThermoFisher).

### Electromobility assay (EMSA)

Nuclear extracts from K562 cells were isolated using the NE-PER Nuclear and Cytoplasmic Extraction Kit (ThermoFisher). 70 base pair sequences containing either the reference alleles for rs2366739 and rs1194196 (referred to as TA) or alternate alleles (referred to as CT) were amplified by PCR using MPRA plasmid library as template. Digoxigenin (DIG) labeled nucleotides (Roche, Basel, Switzerland) were used to create amplified sequences with DIG labeled base pairs. The sequences were purified by agarose gel electrophoresis and the QIAEX II Gel Extraction Kit (Qiagen, Hilden, Germany).

10μg of nuclear extract was incubated with DIG labeled probes in buffer containing 10% glycerol, 20 mM HEPES, 30 mM KCl, 30 mM NaCl, 3 mM MgCl_2_, 1 mM DTT. 1 μg of Poly dI-dC was added to reduce nonspecific binding. The reaction was carried out for 30 mins on ice. The sample was mixed with 10X orange loading dye (Licor, Lincoln, NE) and loaded on 6% acrylamide gel and ran for 4–5 hours with 0.5% TBE buffer. The DNA-protein complex was transferred on positively charged Biodyne nylon membrane (Pall Industries, Fort Washington, NY) using 0.5% TBE for 45 minutes. The membrane was incubated in blocking solution for 30 mins at RT, followed by Anti-Digoxigenin-AP antibody containing blocking solution for 30 mins at RT. The membrane was then washed twice for 15 minutes each using washing solution, visualized using chemiluminescence, and quantified.

### CRISPR-modified cell line generation

To generate genomic deletion mutants, the CRISPR/Cas9 system was used as previously reported [50]. Two guide RNAs (sgRNA) flanking 573 base pairs containing rs2366739 and rs1194196 were designed (IDT DNA). The design of sgRNA pairs for targeting and prediction of off-target sites were based on online tools: CRISPR Design (http://crispr.mit.edu/) and CRISPOR (http://www.crispor.org)[51]. Two guide RNAs, 5'-TACCCCCATTGTATCTATCTAGG-3’ and 5'- CTACAGTAAATACACTTGTCAGG -3’ were used to delete the 573 basepair region.

Pairs of complementary DNA oligos (IDTDNA, Coralville, IA) were individually phosphorylated with T4 polynucleotide kinase (NEB, Ipswitch, MA) and then annealed. Each DNA oligo duplex had 5' overhangs (forward: ACCG, reverse: AAAC) designed to be directly cloned into the BbsI or BsaI-digested and dephosphorylated AIO-GFP(Cas9) vector using the Quick Ligation Kit (NEB). The first and second sgRNA was cloned into the BbsI and BsaI sites, respectively, and confirmed by colony PCR and sequencing. The plasmid was transfected in K562 using Lipofectamine2000 (ThermoFisher) or Meg-01 with Nucleofection.(Lonza, Basel, Switzerland). After 24 hours, the GFP positive cells were sorted by flow cytometry and individually seeded in a 96 well plate. Single colonies were expanded further and a cell line was established from a single clone. Deletion was confirmed by PCR and sequencing.

### Reverse transcription and quantitative real time PCR:

Total RNA was isolated using Trizol Reagent (ThermoFisher). 3μg total RNA was used for first strand cDNA synthesis with the SuperScript III First-Strand Synthesis System (ThermoFisher). To evaluate relative expression levels of mRNAs, we performed qRT-PCR with the Power SYBR Green PCR master mix (Life Technologies, Carlsbad, CA) normalized to Actin. We carried out real time PCR reaction and analyses in 384-well optical reaction plates using the CFX384 instrument (Bio-Rad, Hercules, CA).

## Supporting information

S1 Table Sequences of MPRA controls. (PDF)

S2 Table RNA-seq quality. (PDF)

S3 Table Sequences generated for luciferase assays. (PDF)

S4 Table Complete MPRA results of positive controls. (PDF)

S5 Table Complete MPRA results of CD36 variants. (PDF)

S1 Fig Combined activity scatterplots and posterior plots. (PDF)

S2 Fig Hypothesized mechanism. (PDF)

S3 Fig Plasmid library barcode representation. (PDF)

S4 Fig Kruschke diagram showing the generative model underlying the Bayesian analysis. (PDF)
