# Supplementary material for: Functionalization of CD36 cardiovascular disease and expression associated variants by interdisciplinary high throughput analysis
Source: PLoS Genet. 2019 Jul 25;15(7):e1008287. doi: 10.1371/journal.pgen.1008287 (PMC6684090; doi:10.1371/journal.pgen.1008287)
Supplement: S1 Table — (PDF) [file pgen.1008287.s001.pdf]

Table S1. Complete MPRA results of positive controls

Control	Transcription Shift	t-test		u-test		Bayesian Model	
		P-value	Q-value	P-value	Q-value	Posterior Mean	95% Credible Interval
URUOS	-2.55525	1.94E-131	1.76E-129	2.08E-38	1.89E-36	-2.44298	-2.616 : -2.267
PKRRE5	-1.00354	9.86E-73	4.49E-71	1.41E-37	6.40E-36	-1.11463	-1.282 : -0.944
PKRRE3	-0.87213	1.24E-71	3.77E-70	4.44E-37	1.35E-35	-1.01771	-1.183 : -0.848
ALAS23	-2.08592	8.95E-62	2.04E-60	5.82E-33	8.82E-32	-2.00429	-2.276 : -1.703
ALAS22	-2.413	2.53E-57	3.83E-56	1.11E-28	1.12E-27	-2.48037	-2.838 : -2.114
ALAS21	-2.10623	6.23E-57	8.10E-56	1.52E-32	1.97E-31	-2.00922	-2.33 : -1.699
PKRRE4	-0.89499	2.37E-56	2.70E-55	7.73E-36	1.76E-34	-0.93448	-1.097 : -0.769
PKRRE1	-0.69606	7.38E-39	7.46E-38	2.57E-30	2.92E-29	-0.75478	-0.924 : -0.59
PKRRE2	-0.26871	1.76E-06	1.45E-05	1.47E-08	1.34E-07	-0.37922	-0.698 : -0.055
HBG2	0.06142	4.96E-01	7.28E-01	7.87E-01	8.97E-01	-0.01277	-0.231 : 0.214
