# Supplementary material for: Functionalization of CD36 cardiovascular disease and expression associated variants by interdisciplinary high throughput analysis
Source: PLoS Genet. 2019 Jul 25;15(7):e1008287. doi: 10.1371/journal.pgen.1008287 (PMC6684090; doi:10.1371/journal.pgen.1008287)
Supplement: S2 Table — (PDF) [file pgen.1008287.s002.pdf]

Table S2. Complete MPRA results of CD36 variants.

Control	Transcription Shift	t-test		u-test		Bayesian Model	
		P-value	Q-value	P-value	Q-value	Posterior Mean	95% Credible Interval
rs2366739	-1.04532	6.87E-59	1.25E-57	1.41E-35	2.57E-34	-1.13049	-1.295 : -0.955
rs940542	-0.2785	2.56E-07	2.33E-06	6.51E-07	5.39E-06	-0.29285	-0.481 : -0.12
rs1093831	-0.273	1.07E-04	8.09E-04	7.70E-05	5.84E-04	0.10111	-0.113 : 0.315
rs6467258	0.22024	6.72E-03	4.37E-02	7.47E-04	5.23E-03	-0.05448	-0.259 : 0.16
rs1194196	-0.23346	1.21E-02	6.45E-02	1.07E-03	6.93E-03	-0.08582	-0.271 : 0.111
rs11464747	0.36056	1.02E-03	7.16E-03	1.70E-03	1.03E-02	1.23996	0.962 : 1.518
rs819456	0.31541	3.05E-02	1.32E-01	2.68E-03	1.52E-02	0.46373	0.079 : 0.861
rs6961069	0.21993	1.14E-02	6.45E-02	2.90E-03	1.55E-02	0.01234	-0.211 : 0.233
rs819457	0.18773	1.58E-02	7.58E-02	3.07E-03	1.55E-02	0.14833	-0.072 : 0.372
rs28851188	0.20479	9.63E-03	5.84E-02	1.53E-02	6.94E-02	0.07836	-0.132 : 0.282
rs1761661	-0.48586	1.51E-01	3.70E-01	1.47E-02	6.94E-02	-0.44408	-1.109 : 0.227
rs1608671	0.2257	1.32E-02	6.69E-02	1.83E-02	7.91E-02	0.24244	-0.001 : 0.463
rs13233631	0.23128	6.44E-02	2.17E-01	2.29E-02	9.47E-02	-0.03622	-0.305 : 0.253
rs819442	-0.14388	1.59E-01	3.77E-01	2.45E-02	9.67E-02	0.05905	-0.24 : 0.361
rs819445	-0.01314	9.05E-01	9.89E-01	3.11E-02	1.18E-01	-0.02339	-0.289 : 0.237
rs1761667	-0.18177	4.86E-02	1.84E-01	3.85E-02	1.40E-01	-0.08644	-0.315 : 0.148

rs4626520	0.13624	6.17E-02	2.17E-01	4.96E-02	1.67E-01	0.02326	-0.188 : 0.223
rs2781841	-0.1183	1.50E-01	3.70E-01	4.85E-02	1.67E-01	-0.10839	-0.332 : 0.128
rs1851935	0.46972	2.08E-02	9.44E-02	5.60E-02	1.82E-01	0.58079	0.15 : 0.982
rs2177616	-0.09018	1.68E-01	3.82E-01	6.19E-02	1.94E-01	-0.00484	-0.188 : 0.191
rs1194197	0.11786	1.07E-01	3.03E-01	7.32E-02	2.08E-01	0.12638	-0.085 : 0.338
rs4731643	-0.15098	1.11E-01	3.05E-01	7.11E-02	2.08E-01	-0.11007	-0.36 : 0.128
rs7810280	-0.11062	3.32E-01	6.05E-01	7.19E-02	2.08E-01	-0.22907	-0.463 : 0.009
rs10233710	0.16293	3.71E-02	1.53E-01	8.50E-02	2.34E-01	0.26916	0.051 : 0.485
rs1194179	0.05632	4.24E-01	6.88E-01	9.44E-02	2.53E-01	-0.14227	-0.349 : 0.06
rs1953298	-0.07658	5.12E-01	7.39E-01	1.13E-01	2.95E-01	-0.0912	-0.372 : 0.176
rs1093833	0.54135	1.04E-01	3.03E-01	1.23E-01	3.12E-01	1.00913	0.271 : 1.764
rs2366855	-0.04748	6.02E-01	8.05E-01	1.28E-01	3.15E-01	-0.26947	-0.505 : -0.034
rs12155030	0.49526	3.87E-02	1.53E-01	1.43E-01	3.24E-01	0.21049	-0.404 : 0.784
rs3211821	0.15352	6.21E-02	2.17E-01	1.40E-01	3.24E-01	0.17309	-0.052 : 0.38
rs138369160	0.1948	8.07E-02	2.62E-01	1.46E-01	3.24E-01	0.31868	0.018 : 0.617
rs17154155	-0.13435	8.45E-02	2.65E-01	1.37E-01	3.24E-01	-0.23316	-0.449 : -0.009
rs4728183	0.00832	9.30E-01	9.89E-01	1.50E-01	3.24E-01	0.01875	-0.223 : 0.266
rs34592988	0.07016	4.06E-01	6.73E-01	1.55E-01	3.29E-01	0.0955	-0.119 : 0.308
rs819436	0.11231	3.14E-01	5.95E-01	1.75E-01	3.61E-01	0.16837	-0.111 : 0.458
rs9649532	0.12441	3.66E-01	6.28E-01	1.94E-01	3.92E-01	0.0279	-0.34 : 0.4

rs3211842	0.09516	1.32E-01	3.55E-01	2.17E-01	4.21E-01	0.24792	0.042 : 0.445
rs1194177	-0.01999	8.35E-01	9.75E-01	2.16E-01	4.21E-01	-0.33384	-0.573 : -0.086
rs28854232	-0.01207	8.93E-01	9.89E-01	2.24E-01	4.25E-01	-0.06846	-0.295 : 0.152
rs1761662	-0.10311	2.55E-01	5.51E-01	2.31E-01	4.28E-01	-0.0865	-0.33 : 0.161
rs1049654	-0.12294	1.62E-01	3.77E-01	2.72E-01	4.88E-01	-0.07185	-0.299 : 0.16
rs1851934	0.09629	4.03E-01	6.73E-01	2.73E-01	4.88E-01	0.40852	0.138 : 0.684
rs1320408	0.06631	2.66E-01	5.62E-01	2.85E-01	4.90E-01	-0.0141	-0.207 : 0.173
rs1761645	-0.02737	7.51E-01	9.14E-01	2.85E-01	4.90E-01	-0.23063	-0.459 : -0.012
rs1093829	0.12957	1.48E-01	3.70E-01	3.23E-01	5.16E-01	0.11022	-0.115 : 0.35
rs1761646	0.08586	2.86E-01	5.75E-01	3.10E-01	5.16E-01	0.14086	-0.064 : 0.371
rs4316098	0.07598	4.62E-01	7.08E-01	3.19E-01	5.16E-01	0.10976	-0.145 : 0.356
rs1953299	0.0382	6.67E-01	8.74E-01	3.21E-01	5.16E-01	-0.5241	-0.742 : -0.308
rs9649527	0.06096	4.33E-01	6.92E-01	3.34E-01	5.24E-01	-0.23275	-0.485 : 0.014
rs11770358	0.15989	2.91E-01	5.75E-01	3.44E-01	5.31E-01	0.13013	-0.193 : 0.463
rs1093830	-0.00965	8.35E-01	9.75E-01	3.65E-01	5.53E-01	-0.0652	-0.239 : 0.118
rs819460	0.14181	2.72E-01	5.63E-01	3.72E-01	5.55E-01	0.15343	-0.127 : 0.427
rs1194182	-0.2146	9.11E-02	2.76E-01	4.08E-01	5.89E-01	-0.33051	-0.6 : -0.05
rs1761673	0.03448	7.63E-01	9.14E-01	4.08E-01	5.89E-01	0.11188	-0.133 : 0.356
rs7793698	0.04443	7.36E-01	9.14E-01	4.29E-01	6.09E-01	-0.02528	-0.334 : 0.286
rs13236689	0.09468	3.04E-01	5.89E-01	4.91E-01	6.87E-01	0.03031	-0.207 : 0.262

rs1093834	-0.00567	9.20E-01	9.89E-01	5.05E-01	6.96E-01	0.15143	-0.051 : 0.35
rs6467251	0.17471	4.67E-01	7.08E-01	6.15E-01	8.36E-01	-0.03797	-0.526 : 0.432
rs701269	0.08099	3.24E-01	6.01E-01	6.55E-01	8.46E-01	-0.28239	-0.522 : -0.045
rs34736275	-0.02584	6.72E-01	8.74E-01	6.53E-01	8.46E-01	0.02446	-0.157 : 0.212
rs819455	0.00715	9.37E-01	9.89E-01	6.60E-01	8.46E-01	0.00605	-0.23 : 0.231
rs7794010	-0.00445	9.53E-01	9.89E-01	6.53E-01	8.46E-01	0.24392	0.018 : 0.468
rs1527479	0.15073	2.47E-01	5.49E-01	7.24E-01	8.71E-01	0.13982	-0.109 : 0.389
rs12706912	0.10766	3.43E-01	6.12E-01	6.97E-01	8.71E-01	-0.08802	-0.336 : 0.168
rs1537477	0.02838	5.91E-01	8.02E-01	7.37E-01	8.71E-01	0.05378	-0.139 : 0.237
rs1194178	0.02119	8.72E-01	9.89E-01	7.25E-01	8.71E-01	0.15633	-0.149 : 0.443
rs1054516	-0.0088	8.97E-01	9.89E-01	7.12E-01	8.71E-01	0.01406	-0.188 : 0.212
rs2366744	-0.00327	9.57E-01	9.89E-01	7.36E-01	8.71E-01	-0.09881	-0.285 : 0.093
rs819444	0.03194	6.87E-01	8.80E-01	7.55E-01	8.81E-01	0.02406	-0.266 : 0.319
rs1194195	-0.082	4.59E-01	7.08E-01	8.18E-01	8.97E-01	-0.11836	-0.359 : 0.126
rs1093835	-0.03549	5.52E-01	7.64E-01	8.02E-01	8.97E-01	-0.0405	-0.22 : 0.134
rs3212160	0.04682	5.54E-01	7.64E-01	8.10E-01	8.97E-01	0.01562	-0.196 : 0.232
rs819443	-0.00145	9.89E-01	9.89E-01	8.14E-01	8.97E-01	-0.09974	-0.433 : 0.226
rs6961024	0.0029	9.72E-01	9.89E-01	8.49E-01	9.19E-01	0.26364	0.068 : 0.472
rs10499859	0.09846	3.50E-01	6.13E-01	8.90E-01	9.30E-01	0.16667	-0.075 : 0.423
rs17154155_ALT	-0.04608	4.96E-01	7.28E-01	8.85E-01	9.30E-01	0.08127	-0.122 : 0.284

rs1404312	-0.04521	7.15E-01	9.03E-01	8.72E-01	9.30E-01	-0.19578	-0.454 : 0.064
rs35660939	-0.06731	5.31E-01	7.54E-01	9.58E-01	9.69E-01	-0.09644	-0.37 : 0.167
rs11771152	-0.00454	9.64E-01	9.89E-01	9.48E-01	9.69E-01	-0.00632	-0.267 : 0.256
rs11770907	-0.00199	9.83E-01	9.89E-01	9.37E-01	9.69E-01	0.03896	-0.211 : 0.292
rs9649529	-0.01129	7.63E-01	9.14E-01	9.90E-01	9.90E-01	-0.05748	-0.237 : 0.128
