# Supplementary material for: Functionalization of CD36 cardiovascular disease and expression associated variants by interdisciplinary high throughput analysis
Source: PLoS Genet. 2019 Jul 25;15(7):e1008287. doi: 10.1371/journal.pgen.1008287 (PMC6684090; doi:10.1371/journal.pgen.1008287)
Supplement: S3 Table — (PDF) [file pgen.1008287.s003.pdf]

Table S3: Sequences of MPRA Controls

Gene	Type	Sequence
PKRRE	Ref	AGGAAATGCCAGGAGATGAGGGCAGAGAGCAGGCCGTTCTGGGGGAGGGATTCTGTGGGGACAGGGTGGCCTACTGGGTGTGCCCCTTTTCTCTTCTCTGTCTCCCTTAGATAAGACCAGCAGTTTTGTCATCCTCTCCCTCTCATTCCA
PKRRE	Mut	AGGAAATGCCAGGAGATGAGGGCAGAGAGCAGGCCGTTCTGGGGGAGGGATTCTGTGGGGACAGGGTGGCCTACTGGGTGTGCCCCTTTTCTCTTATCTGTCTCCCTTAGATAAGACCAGCAGTTTTGTCATCCTCTCCCTCTCATTCCA
PKRRE	Ref	AGGAAATGCCAGGAGATGAGGGCAGAGAGCAGGCCGTTCTGGGGGAGGGATTCTGTGGGGACAGGGTGGCCTACTGGGTGTGCCCCTTTTCTCTTCTCTGTCTCCCTTAGATAAGACCAGCAGTTTTGTCATCCTCTCCCTCTCATTCCA
PKRRE	Mut	AGGAAATGCCAGGAGATGAGGGCAGAGAGCAGGCCGTTCTGGGGGAGGGATTCTGTGGGGACAGGGTGGCCTACTGGGTGTGCCCCTTTTCTCTTCGCTGTCTCCCTTAGATAAGACCAGCAGTTTTGTCATCCTCTCCCTCTCATTCCA
PKRRE	Ref	AGGAAATGCCAGGAGATGAGGGCAGAGAGCAGGCCGTTCTGGGGGAGGGATTCTGTGGGGACAGGGTGGCCTACTGGGTGTGCCCCTTTTCTCTTCTCTGTCTCCCTTAGATAAGACCAGCAGTTTTGTCATCCTCTCCCTCTCATTCCA
PKRRE	Mut	AGGAAATGCCAGGAGATGAGGGCAGAGAGCAGGCCGTTCTGGGGGAGGGATTCTGTGGGGACAGGGTGGCCTACTGGGTGTGCCCCTTTTCTCTTCTATGTCTCCCTTAGATAAGACCAGCAGTTTTGTCATCCTCTCCCTCTCATTCCA
PKRRE	Ref	AGGAAATGCCAGGAGATGAGGGCAGAGAGCAGGCCGTTCTGGGGGAGGGATTCTGTGGGGACAGGGTGGCCTACTGGGTGTGCCCCTTTTCTCTTCTCTGTCTCCCTTAGATAAGACCAGCAGTTTTGTCATCCTCTCCCTCTCATTCCA
PKRRE	Mut	AGGAAATGCCAGGAGATGAGGGCAGAGAGCAGGCCGTTCTGGGGGAGGGATTCTGTGGGGACAGGGTGGCCTACTGGGTGTGCCCCTTTTCTCTTCTCGGTCTCCCTTAGATAAGACCAGCAGTTTTGTCATCCTCTCCCTCTCATTCCA
PKRRE	Ref	AGGAAATGCCAGGAGATGAGGGCAGAGAGCAGGCCGTTCTGGGGGAGGGATTCTGTGGGGACAGGGTGGCCTACTGGGTGTGCCCCTTTTCTCTTCTCTGTCTCCCTTAGATAAGACCAGCAGTTTTGTCATCCTCTCCCTCTCATTCCA
PKRRE	Mut	AGGAAATGCCAGGAGATGAGGGCAGAGAGCAGGCCGTTCTGGGGGAGGGATTCTGTGGGGACAGGGTGGCCTACTGGGTGTGCCCCTTTTCTCTTCTCTCTCTCCCTTAGATAAGACCAGCAGTTTTGTCATCCTCTCCCTCTCATTCCA
ALAS2	Ref	CTGGAGATATGTGTTTCCCTTCCCCTGCCTGCTTGTGAAAGCTAAAGCACTTGGGGCTGAGCCTGCAGACCACAGATAAAGTTGCCAGAGTTTATCGCCATTGGGGTCTGACCACTCCCCAAGCTGAGACCAGGGCGTGGGAGAGAAGAG
ALAS2	Mut	CTGGAGATATGTGTTTCCCTTCCCCTGCCTGCTTGTGAAAGCTAAAGCACTTGGGGCTGAGCCTGCAGACCACACATAAAGTTGCCAGAGTTTATCGCCATTGGGGTCTGACCACTCCCCAAGCTGAGACCAGGGCGTGGGAGAGAAGAG
ALAS2	Ref	TGGAGATATGTGTTTCCCTTCCCCTGCCTGCTTGTGAAAGCTAAAGCACTTGGGGCTGAGCCTGCAGACCACAGATAAAGTTGCCAGAGTTTATCGCCATTGGGGTCTGACCACTCCCCAAGCTGAGACCAGGGCGTGGGAGAGAAGAGG
ALAS2	Mut	TGGAGATATGTGTTTCCCTTCCCCTGCCTGCTTGTGAAAGCTAAAGCACTTGGGGCTGAGCCTGCAGACCACAGGTAAAGTTGCCAGAGTTTATCGCCATTGGGGTCTGACCACTCCCCAAGCTGAGACCAGGGCGTGGGAGAGAAGAGG
ALAS2	Ref	GGAGATATGTGTTTCCCTTCCCCTGCCTGCTTGTGAAAGCTAAAGCACTTGGGGCTGAGCCTGCAGACCACAGATAAAGTTGCCAGAGTTTATCGCCATTGGGGTCTGACCACTCCCCAAGCTGAGACCAGGGCGTGGGAGAGAAGAGGA
ALAS2	Mut	GGAGATATGTGTTTCCCTTCCCCTGCCTGCTTGTGAAAGCTAAAGCACTTGGGGCTGAGCCTGCAGACCACAGACAAAGTTGCCAGAGTTTATCGCCATTGGGGTCTGACCACTCCCCAAGCTGAGACCAGGGCGTGGGAGAGAAGAGGA
UROS	Ref	TGGAAGAGACCTATCCCTCCTACACCATGACTTAGCACTAATGGGCTTGTTCTTTCTGAAGACCCCTGTCACTGATAAGGCCAAGAAAGAGCATGTTAGCAGTTGATATCACTTGGAAAGACAGATGAAACCATTAGTGAAAGCCAAGTG

UROS	Mut	TGGAAGAGACCTATCCCTCCTACACCATGACTTAGCACTAATGGGCTTGTTCTTTCTGAAGACCCCTGTCACTGGTAAGG<br>CCAAGAAAGAGCATGTTAGCAGTTGATATCACTTGGAAAGACAGATGAAACCATTAGTGAAAGCCAAGTG
SPI1	Ref	GTTGTTTAAAATTTTCTCTGAGACACGACGTTCTCCTACCTAACTCTTATTGAAATGAACAAAAAACCCTCCTCCAATTACC<br>CACCCTCCCCACTCTCCCCTCTAGACTAAATGTATGCCTGAACTCTGAGGGTTCCGTCATGGGGCCCA
SPI1	Mut	GTTGTTTAAAATTTTCTCTGAGACACGACGTTCTCCTACCTAACTCTTATTGAAATGAACAAAAAACCCTCCTCTAATTACC<br>CACCCTCCCCACTCTCCCCTCTAGACTAAATGTATGCCTGAACTCTGAGGGTTCCGTCATGGGGCCCA
HBG2	Ref	TAACATTAATCTATTCCTGCACTGAAACTGTTGCTTTATAGGATTTTTCACTACACTAATGAGAACTTAAGAGATAATGGCC<br>TAAAACCACAGAGAGTATATTCAAAGATAAGTATAGCACTTCTTATTTGGAAACCAATGCTTACTAAA
HBG2	Mut	TAACATTAATCTATTCCTGCACTGAAACTGTTGCTTTATAGGATTTTTCACTACACTAATGAGAACTTAAGAGAGAATGGC<br>CTAAAACCACAGAGAGTATATTCAAAGATAAGTATAGCACTTCTTATTTGGAAACCAATGCTTACTAAA
