# Supplementary material for: Functionalization of CD36 cardiovascular disease and expression associated variants by interdisciplinary high throughput analysis
Source: PLoS Genet. 2019 Jul 25;15(7):e1008287. doi: 10.1371/journal.pgen.1008287 (PMC6684090; doi:10.1371/journal.pgen.1008287)
Supplement: S4 Table — (PDF) [file pgen.1008287.s004.pdf]

Table S4: RNA-Seq Quality

Sample	Sample Depth	Number of Reads	Fraction Usable Reads	Fraction 0-count barcodes
cDNA1	1891321	2739571	0.69	0.09
cDNA2	2967012	4109045	0.72	0.09
cDNA3	1577854	2242279	0.70	0.12
Plasmid1	1968644	2757765	0.71	0.07
Plasmid2	3023219	4342543	0.70	0.06
