# Supplementary material for: Functionalization of CD36 cardiovascular disease and expression associated variants by interdisciplinary high throughput analysis
Source: PLoS Genet. 2019 Jul 25;15(7):e1008287. doi: 10.1371/journal.pgen.1008287 (PMC6684090; doi:10.1371/journal.pgen.1008287)
Supplement: S5 Table — (PDF) [file pgen.1008287.s005.pdf]

Table S5. Sequences generated for luciferase assays.

SNP-genotype	Sequence*
rs1093833 T (Ref)	GCAGCAGCCACAACCAAGTGTTAGTGGGTATAGGGGTGCC
rs1093833 C (Alt)	GCAGCAGCCACAACCAAGTGCTAGTGGGTATAGGGGTGCC
rs7810280 G (Ref)	AATCAACCAAGATGATTTACGGGTCAAAGTATTATAGTGT
rs7810280 A (Alt)	AATCAACCAAGATGATTTACAGGTCAAAGTATTATAGTGT
rs819456 T (Ref)	TGCTGTTCTTGTAATAGTGATTGGGTCTCATGAAATCTGA
rs819456 A (Alt)	TGCTGTTCTTGTAATAGTGAATGGGTCTCATGAAATCTGA
rs940542T (Ref)	TTTAACTGAATTTTTAATGTTGTTAACTGAGATAAGTGAA
Rs940542 C (Alt)	TTTAACTGAATTTTTAATGTCGTTAACTGAGATAAGTGAA
rs2366739+rs1194196 TA (Ref)	TCCCATGCTGTTCTTGTAATAGTGATTGGGTCTCATGAAATCTGATGTTTTTAAAAACGG GAGTTTCTCTGAACAGTCTC
rs2366739+rs1194196 CT (Alt)	TCCCATGCTGTTCTTGTAATAGTGACTGGGTCTCATGAAATCTGATGTTTTTATAAACGG GAGTTTCTCTGAACAGTCTC

*Variant is in red
