# Supplementary material for: Functionalization of CD36 cardiovascular disease and expression associated variants by interdisciplinary high throughput analysis
Source: PLoS Genet. 2019 Jul 25;15(7):e1008287. doi: 10.1371/journal.pgen.1008287 (PMC6684090; doi:10.1371/journal.pgen.1008287)
Supplement: S1 Fig — (PDF) [file pgen.1008287.s006.pdf]

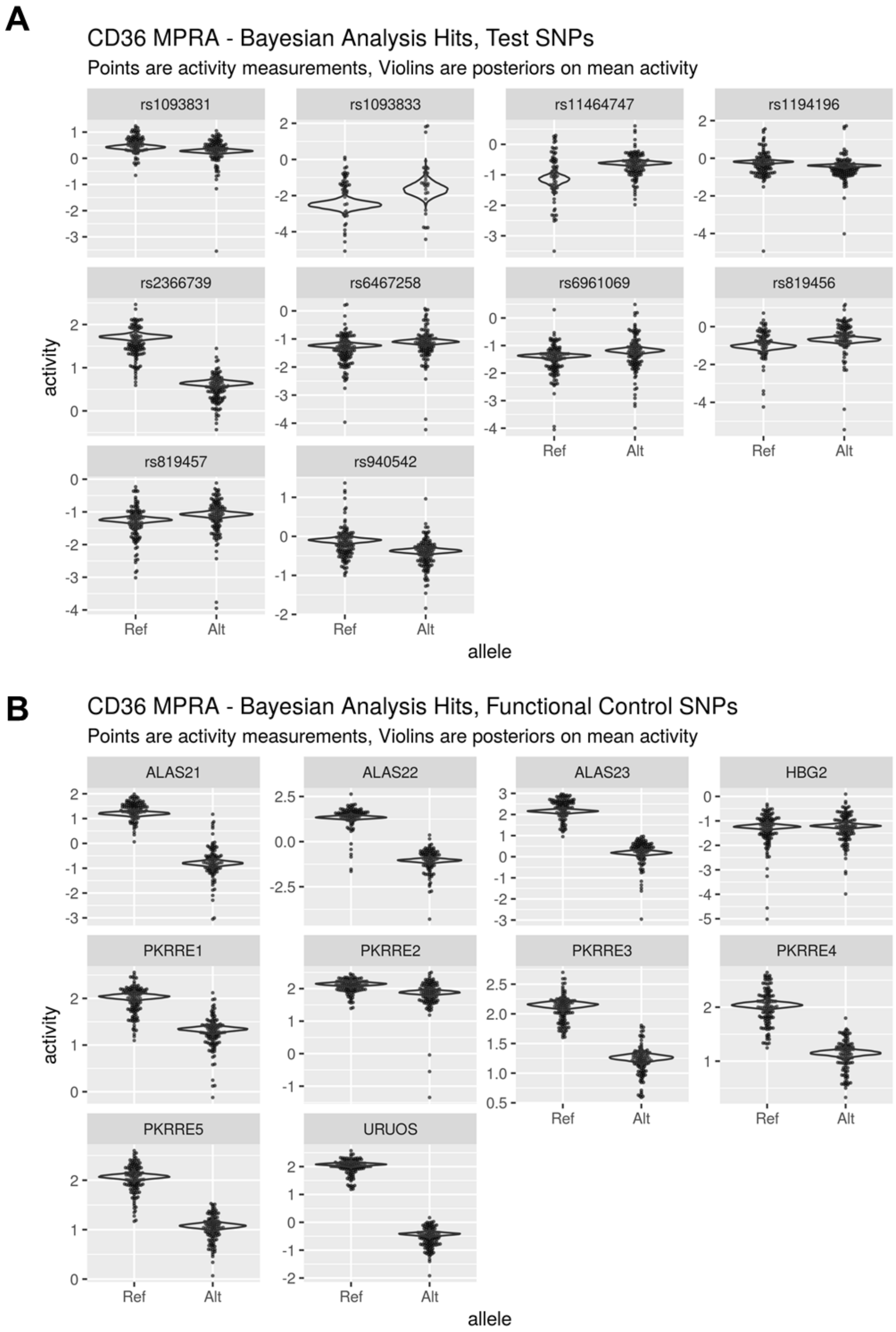

Figure S1 – Combined activity scatterplots and posterior plots: Points show activity measurements for individual barcodes, while violins show the posterior on the mean activity level for each allele from the Bayesian model for (A) the detected functional test SNPs and (B) the Controls.
