# Supplementary material for: Functionalization of CD36 cardiovascular disease and expression associated variants by interdisciplinary high throughput analysis
Source: PLoS Genet. 2019 Jul 25;15(7):e1008287. doi: 10.1371/journal.pgen.1008287 (PMC6684090; doi:10.1371/journal.pgen.1008287)
Supplement: S2 Fig — (PDF) [file pgen.1008287.s007.pdf]

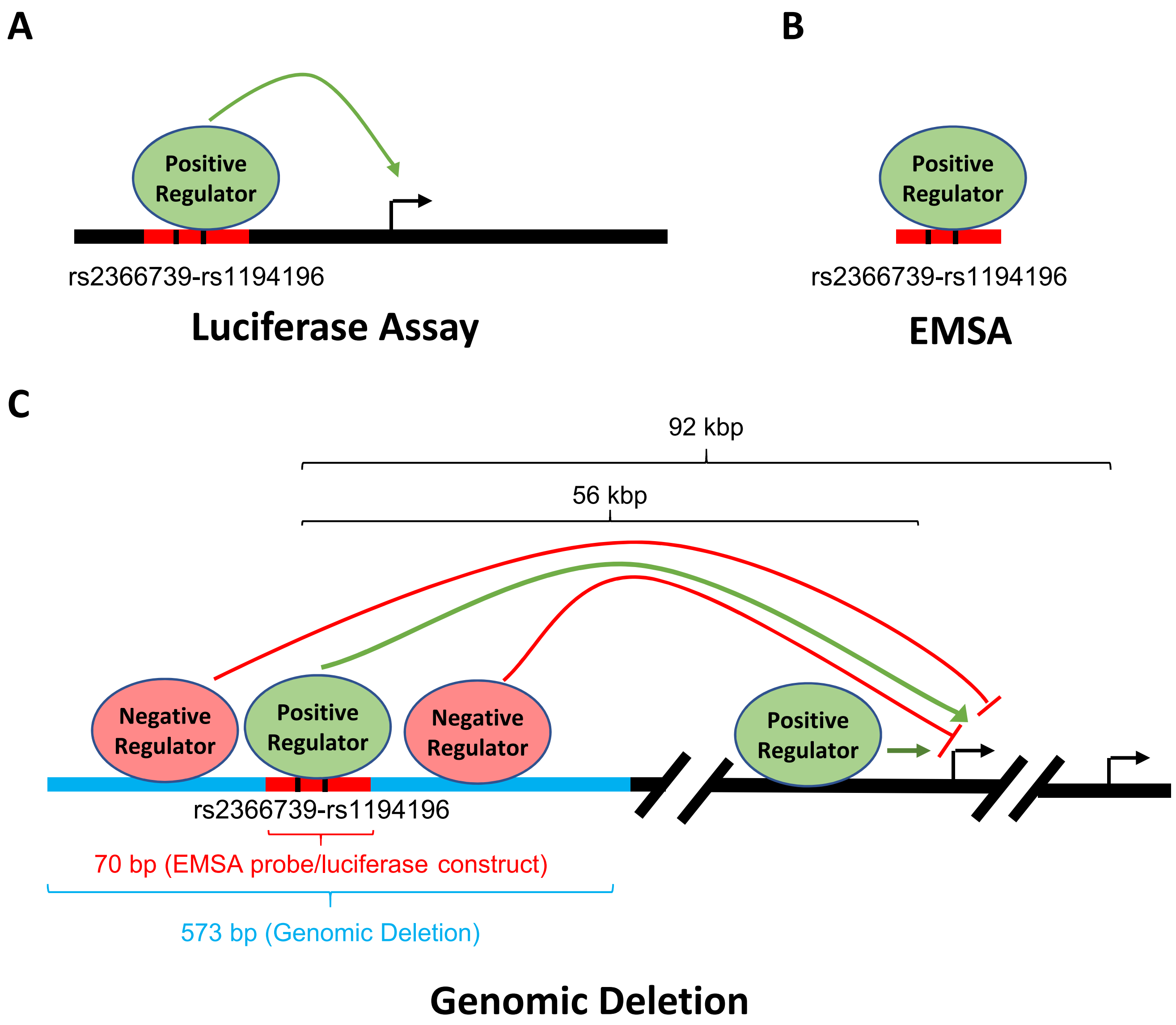

Figure S2. – Hypothesized Mechanism. 70bp genomic fragment used in (A) Luciferase and (B) Electromobility Shift Assay (EMSA) demonstrate the binding of a positive regulatory factor to the rs2366739-rs1194196 locus. (C) In a genomic context, deletion of the 573bp fragment results in the loss of negative regulators and enhanced transcription of *CD36*.
