# Supplementary material for: Functionalization of CD36 cardiovascular disease and expression associated variants by interdisciplinary high throughput analysis
Source: PLoS Genet. 2019 Jul 25;15(7):e1008287. doi: 10.1371/journal.pgen.1008287 (PMC6684090; doi:10.1371/journal.pgen.1008287)
Supplement: S3 Fig — (PDF) [file pgen.1008287.s008.pdf]

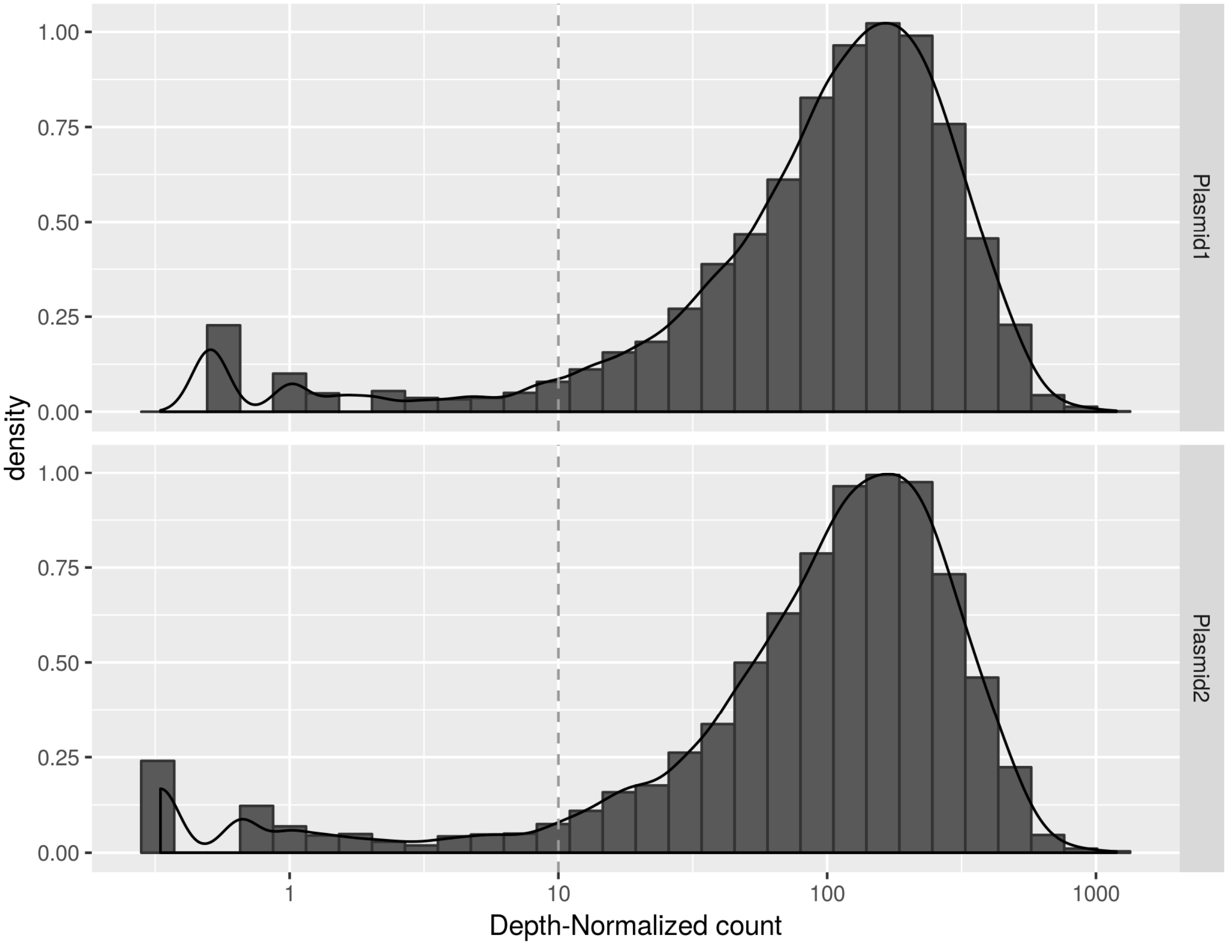

Figure S3 – Plasmid library barcode representation. A (A) histogram and (B) density function showing the abundance of DNA barcodes in the plasmid libraries. Barcodes present at very low levels in the plasmid library will cause the RNA output to be highly variable. A minimum plasmid library abundance is chosen by inspection to use as a cutoff. Barcodes that are not present above this level will be discarded from the downstream analysis. The counts are first normalized for sequencing depth by dividing through by the respective sample number of reads and multiplying by one million. A dashed line indicates the selected depth-normalized count cutoff.
