# Supplementary material for: Functionalization of CD36 cardiovascular disease and expression associated variants by interdisciplinary high throughput analysis
Source: PLoS Genet. 2019 Jul 25;15(7):e1008287. doi: 10.1371/journal.pgen.1008287 (PMC6684090; doi:10.1371/journal.pgen.1008287)
Supplement: S4 Fig — (PDF) [file pgen.1008287.s009.pdf]

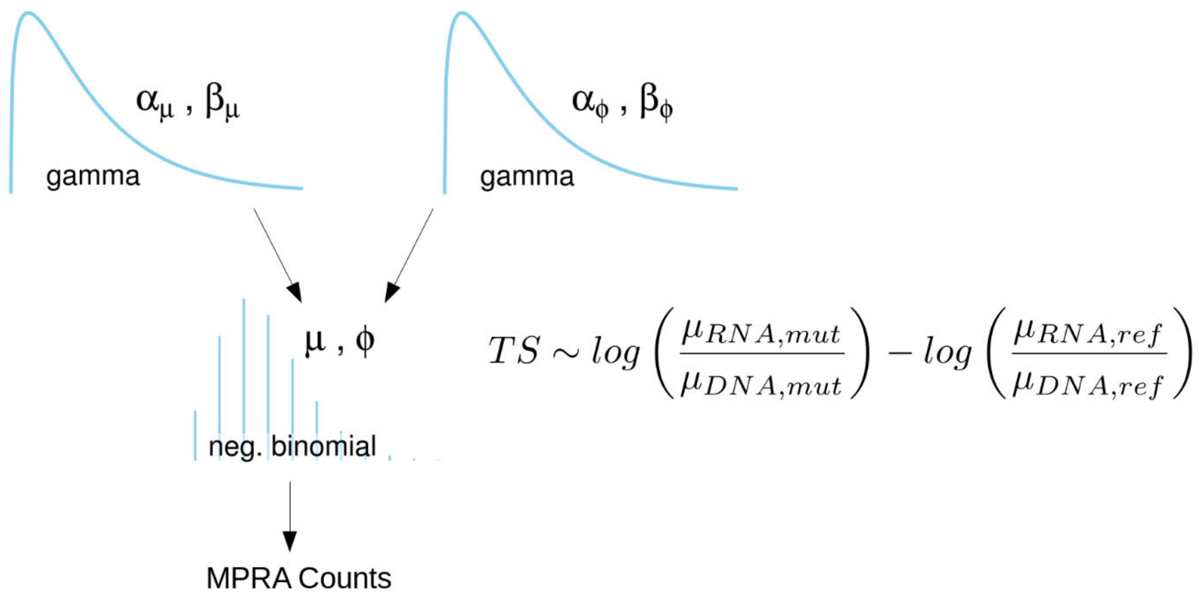

Figure S4 – Kruschke diagram showing the generative model underlying the Bayesian analysis. The mean parameters for the separate alleles and nucleic acids are aggregated after fitting the model into a posterior on transcription shift that is used to identify functional SNPs.
